# An Innovative Multi-Omics Model Integrating Latent Alignment and Attention Mechanism for Drug Response Prediction

**DOI:** 10.3390/jpm14070694

**Published:** 2024-06-27

**Authors:** Hui-O Chen, Yuan-Chi Cui, Peng-Chan Lin, Jung-Hsien Chiang

**Affiliations:** 1 Department of Computer Science and Information Engineering, National Cheng Kung University, Tainan 701, Taiwan; 2 Institute of Medical Informatics, National Cheng Kung University, Tainan 701, Taiwan; 3 Department of Oncology, National Cheng Kung University Hospital, College of Medicine, National Cheng Kung University, Tainan 701, Taiwan; 4 Department of Genomic Medicine, National Cheng Kung University Hospital, College of Medicine, National Cheng Kung University, Tainan 701, Taiwan

**Keywords:** multi-omics, drug response, latent alignment, deep learning, attention module

## Abstract

By using omics, we can now examine all components of biological systems simultaneously. Deep learning-based drug prediction methods have shown promise by integrating cancer-related multi-omics data. However, the complex interaction between genes poses challenges in accurately projecting multi-omics data. In this research, we present a predictive model for drug response that incorporates diverse types of omics data, comprising genetic mutation, copy number variation, methylation, and gene expression data. This study proposes latent alignment for information mismatch in integration, which is achieved through an attention module capturing interactions among diverse types of omics data. The latent alignment and attention modules significantly improve predictions, outperforming the baseline model, with MSE = 1.1333, F1-score = 0.5342, and AUROC = 0.5776. High accuracy was achieved in predicting drug responses for piplartine and tenovin-6, while the accuracy was comparatively lower for mitomycin-C and obatoclax. The latent alignment module exclusively outperforms the baseline model, enhancing the MSE by 0.2375, the F1-score by 4.84%, and the AUROC by 6.1%. Similarly, the attention module only improves these metrics by 0.1899, 2.88%, and 2.84%, respectively. In the interpretability case study, panobinostat exhibited the most effective predicted response, with a value of −4.895. We provide reliable insights for drug selection in personalized medicine by identifying crucial genetic factors influencing drug response.

## 1. Introduction

Cancer continues to be a significant cause of mortality worldwide, with a high mortality rate that demands urgent attention. The close association between cancer and gene alterations has been well established, arising from both intrinsic and extrinsic factors [1,2]. Targeted therapy has emerged as a potential solution for cancer treatment, but the genetic heterogeneity of cancer poses challenges, leading to diverse responses among patients. The integration of multi-omics data, including genomics, epigenomics, and transcriptomics, has shown promise in predicting drug responses for cancer treatment.

Deep learning models have proven effective in extracting essential features from multi-omics data to predict drug responses [3,4,5,6]. For instance, Chiu et al. [7] proposed using different deep neural networks to integrate gene expression and gene mutation data and then utilizing the integrated features for drug response prediction. Building on this approach, Sharifi-Noghabi, H. et al. [8] introduced the multi-omics late integration (MOLI) method, which further enriches multi-omics data and employs contrastive loss to enhance the model performance. However, two aspects of these methods have not been considered. One overlooked aspect is the absence of consideration of genomics biology, including the central dogma [9]. In molecular biology, the central dogma elucidates how genetic information moves through a biological system, illustrating the coordinated interplay of DNA, RNA, and proteins [10]. The other aspect is their ineffectiveness in proficiently capturing correlations within inter-omics data, which play a crucial role in accurately predicting drug response [11].

Intricate interplay, such as that of the genomics flow from DNA to DNA or DNA to RNA among different omics data sources, must be considered during integration to improve prediction performance. A challenge arises during integration, as different omics datasets undergo dimensionality transformation without considering information from one another, resulting in feature misalignment [12] in the latent space. This misalignment hinders the integration results of the model and poses a critical problem to address.

In addition, to capture the correlation between different types of omics data, some studies have proposed using an attention mechanism. For example, Wang et al. [13] proposed a model that integrates multi-omics data to increase the richness of the input. An attention layer was introduced to capture the inter-omics correlations and assign importance weights to different features. Thus, an attention mechanism allows for more effective information sharing among different omics features, resulting in a more comprehensive understanding of the data.

To overcome these obstacles, this study aims to investigate the correlation between genomics-related multi-omics data and cancer. Various types of multi-omics data, such as mutations, copy number variations (CNVs), methylation patterns, and gene expressions specific to cancer cells, will be utilized to predict drug response concentrations across different cell line samples. To address the issue of information misalignment during integration, the study proposes a method called latent alignment, which aligns multi-omics data into a common latent space after feature extraction. Additionally, an attention module will be implemented to enhance the modeling of interactions between different types of omics data. By successfully addressing the challenges in multi-omics data integration, this research aims to contribute to the advancement of precision medicine in cancer therapeutics.

## 2. Materials and Methods

### 2.1. Study Design and Workflow

**Figure 1:**
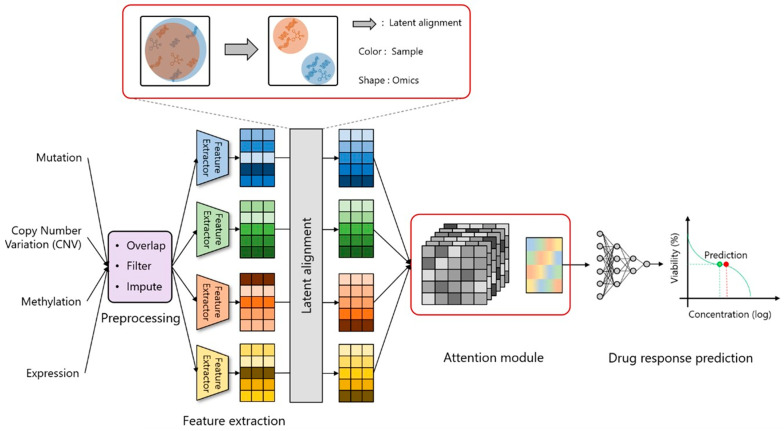
Study design and framework for drug response prediction. We propose a drug response prediction model that integrates multi-omics data (gene expression, mutation, CNV, and methylation data). The model involves data preprocessing and a feature extractor for omics data features and incorporates latent alignment and attention modules.

The Cancer Dependency Map (DepMap) datasets [14] include drug response and diverse cancer-related omics data from cell lines among various cancer types. In this study, we proposed a drug response prediction model that integrates mutation, CNV, methylation, and gene expression data from the DepMap datasets. The model includes data preprocessing and a feature extractor for essential omics data features and integrates a latent alignment and attention module (Figure 1). Multi-omics integration includes calculating the sample similarity to correct latent space misalignment. To improve the prediction model, an attention module was introduced after the latent alignment module (Supplementary Figure S1) to capture relationships among various omics data. A regression loss function was utilized to quantify the disparity between the predicted and actual values, enhancing the precision of drug response concentration prediction.

We constructed a deep learning model with a dense embedding layer of size 1024 for all datasets. The attention module had a dimension of 40, and the output layer dimension was set to 31. To prevent overfitting, a dropout rate of 0.2 was applied to each feature extractor. During the training process, we utilized a learning rate of 10–5 for the feature extractor and 10–6 for the predictor to optimize the model performance. The training was conducted on an Nvidia GTX 1080-Ti GPU to accelerate the computations. To assess the model performance and generalizability, we employed a threefold validation approach. The dataset was divided into three subsets, and the model was trained and evaluated three times, each time with a different subset as the validation set.

We conducted extensive training for 200 epochs to allow the model to converge and capture complex patterns within the data. By utilizing a substantial number of epochs, we aimed to enhance the model predictive capacity.

### 2.2. Datasets and Data Preprocessing

We utilized the DepMap dataset (https://depmap.org/portal/, accessed on 10 Augest 2022) for experiments. We proposed a drug response prediction model, illustrated in Figure 1, that integrates multi-omics information to forecast drug response and uncover underlying genomic factors. The drug response dataset includes IC50(log) values, indicating the drug concentration needed to inhibit 50% of cancer cells. First, we identified all the samples present in the datasets to confirm the availability of gene information from multiple omics datasets. Supplementary Table S1 shows dataset sizes before and after preprocessing, with all datasets containing 543 samples post-preprocessing. The number of genes for multi-omics datasets were 21,840 for CNV, 19,412 for methylation, 19,144 for expression, and 223 for mutation. The mutation, CNV, and expression dataset contains the mutation status (with or without mutations), the copy number (as an integer), and the normalized read count for each gene and each cell line, respectively. The methylation dataset includes the methylation status as a number between 0 and 1 for each CpG site and each cell line. The drug response dataset includes 31 drugs as prediction targets.

To prevent biased model predictions, we excluded samples with missing values exceeding 30% and removed genes with all zeros in each dataset. The remaining missing values were imputed using the mean of the original gene data. These steps yielded complete datasets, minimizing bias and ensuring robust analysis while preserving biological relevance.

### 2.3. Feature Extractor

Since not every gene in the multi-omics data carries equal importance for the final prediction, we employed different feature extractors to capture the essential features from each multi-omics dataset. Equation (1) outlines the learning process of each feature extractor, where **z**^(*i*)^ represents the result of feature extraction for the *i*-th multi-omics dataset, denoted as **x**^(*i*)^.

(1) $$z^{\left(i\right)}=\mathrm{ReLU}(W_{2}^{\left(i\right)}\;(\mathrm{ReLU}(W_{1}^{\left(i\right)}\;x^{\left(i\right)}+b_{1}^{\left(i\right)}))+b_{2}^{\left(i\right)}),\;i=1,\;2,\;3,\;4,$$

where $W_{1}^{\left(i\right)}$ ∈ $R^{d_{1}\times g^{\left(i\right)}}$, $W_{2}^{\left(i\right)}$∈ $R^{d_{2}\times d_{1}}$, $b_{1}^{\left(i\right)}$ ∈ $R^{d_{1}\times bs}$, and $b_{2}^{\left(i\right)}$∈ $R^{d_{2}\times bs}$ mean the weight and bias of each omics dataset at different layers. **d**_1_, **d**_2_ is the dimension of the latent vectors, $g^{\left(i\right)}$ is the number of genes in the *i*-th multi-omics dataset, and bs represents the batch size during training. By leveraging these feature extraction techniques, we can identify and capture the relevant information from each multi-omics dataset.

### 2.4. Latent Alignment

We obtain the sample similarity **S**_*i*,*j*_ by calculating the inner product between different multi-omics features after feature extraction, as shown in Supplementary Figure S1, enabling us to capture the differences between each omics dataset in the same sample in the latent space.

The formula for calculating the sample similarity is shown as follows:

(2) $$S_{i,j}={z^{\left(i\right)}}^{T}\cdot z^{\left(j\right)},\;1\leq i\leq 4,\;1\leq j\leq 4.$$

where *i* and *j* represent the *i*-th and *j*-th omics dataset, respectively.

Ideally, we expect the highest similarity between samples of different omics datasets to occur when they represent the same sample. However, due to the independent nature of the feature extraction process for each omics dataset, the actual sample similarity between different data types in the latent space may deviate from the expected value. To address this disparity, we introduce a learned target T, which represents the average sample similarity among the same omics data features. The formulation of the learned target is defined as

(3) $$T=\mathrm{softmax}(\frac{1}{4\cdot \tau }\cdot (S_{1,1}+S_{2,2}+S_{3,3}+S_{4,4})),$$

where *τ* is a hyperparameter used to regulate the value range of the similarity between different samples.

### 2.5. Attention Module

Supplementary Figure S2 illustrates the architecture of the attention module. The module incorporates information exchange between omics datasets through learnable matrices **M**_*i*,*j*_ to learn the affinity matrices **F**_*i*,*j*_ for each pair of omics datasets. This is accomplished using the following formulas:

(4) $$F_{i,j}=\tanh({z^{\left(j\right)}}^{T}\cdot M_{i,j}\cdot z^{\left(i\right)}),1\;\leq \;i\leq \;4,1\;\leq \;j\leq \;4,\;i\;\neq \;j,$$

where **M**_*i*,*j*_ ∈ $R^{d_{2}\times d_{2}}$.

To integrate this relational information, in Equation (5), ${H^{z}}^{\left(i\right)}$ represents the result of each omics dataset in acquiring information from other omics datasets.

(5) $${H^{z}}^{\left(i\right)}=\tanh(W_{z^{\left(i\right)}}\cdot z^{\left(i\right)}+\Sigma_{j=1,j\neq i}^{4}W_{z^{\left(j\right)}}z^{\left(j\right)}\cdot F_{i,j}),\;1\leq i\leq 4,$$

where $W_{z^{\left(i\right)}}$ ∈ $R^{k\times d_{2}}$ and *k* is the dimension of the latent vectors.

Then, the attention weight $a^{z^{\left(i\right)}}$ of other omics datasets given to the individual omics dataset is obtained through $H^{z^{\left(i\right)}}$, as follows:

(6) $$a^{z^{\left(i\right)}}=\mathrm{softmax}(W_{H^{z^{\left(i\right)}}}\cdot H^{z^{\left(i\right)}}),\;1\leq i\leq 4,$$

where $W_{H^{z^{\left(i\right)}}}$ ∈ $R^{1\times k}$.

Finally, the weight $a^{z^{\left(i\right)}}$ is multiplied by the original feature vector **z**^(*i*)^ and concatenated to obtain the features with the correlation information of different omics datasets,

(7) $${z^{\left(i\right)}}^{\prime }={a^{z}}^{\left(i\right)}\cdot z^{\left(i\right)},\;1\leq i\leq 4,$$

(8) $$Z^{\prime }=\mathrm{concat}({z^{\left(1\right)}}^{\prime },{z^{\left(2\right)}}^{\prime },{z^{\left(3\right)}}^{\prime },{z^{\left(4\right)}}^{\prime }).$$

Next, the integrated features are used to predict drug response, and the prediction can be written as:

(9) $$\mathrm{Pred}=W_{5}(\mathrm{ReLU}(W_{4}(\mathrm{ReLU}(W_{3}\cdot Z^{\prime }+b_{3}))+b_{4}))+b_{5},$$

where **W**_3_ ∈ $R^{d_{3}\times ({4\cdot d}_{2})}$, **W**_4_ ∈ $R^{d_{4}\times d_{3}}$, **W**_5_ ∈ $R^{D\times d_{4}}$, **b**_3_ ∈ $R^{d_{3}\times bs}$, **b**_4_ ∈ $R^{d_{4}\times bs}$, **b**_5_ ∈ $R^{D\times bs}$, **d**_3_, **d**_4_ is the dimension of the latent vectors, and *D* represents the number of drugs to be predicted.

### 2.6. Loss Function

The objective function is

(10) $$L_{\mathrm{reg}}=\frac{1}{N}\left(\sum_{i=1}^{N}\left(y_{i}-{\hat{y}}_{i}\right)\right),$$

where *N* is the number of training samples, $y_{i}$ represents the actual drug response concentration, and ${\hat{y}}_{i}$ is the predicted drug response concentration.

In the latent alignment module, we updated the feature extractors of different omics datasets by calculating the similarity between different features in the latent space and the difference from the target *T*. Our goal was to minimize the difference between the same samples.

(11) $$L_{la}=\Sigma_{i=1}^{3}(\Sigma_{j=i+1}^{4}(\frac{1}{2}\cdot (CE(S_{i,j},T)+CE(S_{j,i},T)))),$$

where *CE*( ) is the cross-entropy function.

The objective function of the model, which incorporates these integration optimization strategies for drug response prediction, can be expressed as follows:

(12) $$L_{total}=\gamma_{r}\times L_{reg}+\gamma_{l}\times L_{la},$$

where *γ_r_* and *γ_l_* are the ratios for these two losses. Here, we set the ratio to 1:1.

### 2.7. Evaluation Metrics

The mean squared error (MSE) is a metric used to quantify the average squared difference between predicted values and actual values. In addition, to assess the effectiveness of individual drugs on patients in the drug concentration dataset, we followed the approach described by Emdadi et al. [15]. Specifically, the F1-score and AUROC were employed as metrics to evaluate the model performance based on the criterion of the median of individual drug response concentrations in the training dataset.

## 3. Results

### 3.1. The Performance of Our Method for Predicting Drug Response

We compared the performance of our model with that of other models [8,13] that also utilized multi-omics data for predicting drug response. To facilitate an unbiased comparison, we utilized consistent datasets and preprocessing methods while implementing the model architectures as detailed in the referenced studies. Table 1 presents the performance of different model architectures in predicting drug response concentrations using multi-omics data.

Table 1 presents the mean and standard deviation values obtained from 3-fold cross-validation. Our model achieved superior prediction performance, yielding the lowest mean squared error (MSE) of 1.1333. Although the MSE difference of 0.1122 between our model and the second-best performing model proposed by Wang, C. et al. [10] (1.2455) may seem slight, notable discrepancies emerge in other metrics. Our model outperforms that of Wang et al. [10], with a 2.27% higher F1-score and a 4.99% improvement in the area under the receiver operating characteristic curve (AUROC).

Supplementary Table S2 presents the drugs ranked by MSE based on the predicted drug response concentrations using our model. The table illustrates MSE variations among different drugs, indicating disparities in the prediction accuracy. Our model exhibits high prediction accuracy for drugs such as piplartine and tenovin-6 but comparatively lower accuracy for mitomycin-C and obatoclax.

### 3.2. Results of Different Combinations of Multi-Omics Datasets

In this experiment, our aim was to investigate how various combinations of omics datasets impact the accuracy of predicting drug response concentrations. We employed a consistent model architecture, varying the input by integrating subsets of omics data, and the results of drug response concentration predictions are presented in Table 2. Among various omics dataset combinations, it is clear that methylation and expression datasets are pivotal in predicting drug response concentrations. The model performance is significantly improved by including these two types of omics data. When employing the CNV and mutation datasets in the experiments, the MSE, F1-score, and AUROC values are 1.3209, 51.11%, and 52.21%, respectively. In contrast, by utilizing methylation and expression datasets, the values improved by 1.2090, 51.04%, and 56.64%, respectively. Despite a marginal 0.07% decrease in the F1-score, there are notable individual enhancements of 0.1119 in the MSE and 4.43% in the AUROC. This result indicates that the methylation and expression datasets are highly relevant in predicting drug response concentrations.

**Figure 2:**
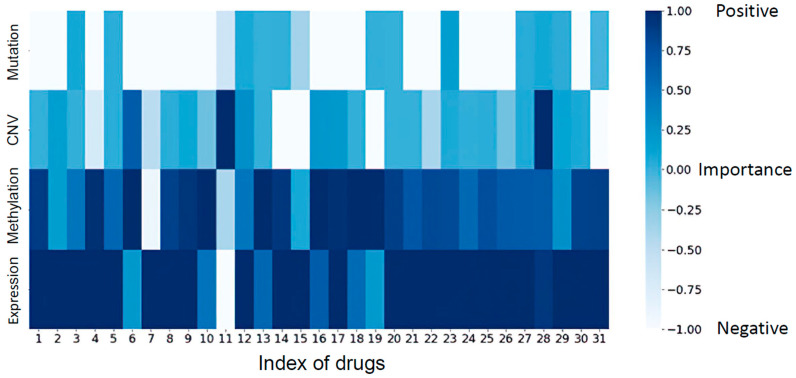
Drug response prediction using different combinations of omics datasets. The *x*-axis represents the index of drugs in the dataset. The *y*-axis represents different omics datasets, and the darkness of the color indicates the impact of the respective omics dataset on the drug response prediction. Darker colors indicate a positive influence, while lighter colors indicate a negative influence on the model predictions. The methylation and gene expression datasets make a meaningful contribution to predicting drug response.

To assess the impact of various omics datasets on 31 drugs, we established the initial prediction results as the baseline. Subsequently, we applied a mask, setting the input of one type of omics dataset to zero, and reperformed drug response concentration prediction with the modified inputs. Based on the new prediction results, we calculated the differences between each drug response and the baseline result to evaluate the impact of excluding a specific type of omics data on the prediction outcomes. If the new prediction result was worse than the baseline, it indicated a positive impact of the omitted omics data on the model predictions. Conversely, if the new prediction result was better than the baseline, it indicated a negative impact. Using this approach, we generated Figure 2, which summarizes the obtained results.

In Figure 2, the *x*-axis represents the index of 31 drugs in the dataset, corresponding to the information provided in Supplementary Table S3. From the chart, it can be observed that the performance of the methylation and expression datasets in predicting drug response concentrations is mostly positive, except for a few drugs such as KIN001-204 and obatoclax, where a negative impact is observed. On the other hand, for the mutation and CNV datasets, a negative influence is found. This observation aligns with the notion mentioned in our previous experiment that the methylation and expression datasets make a meaningful contribution to predicting drug response.

### 3.3. Ablation Experiments Focus on the Latent Alignment and Attention Modules

In ablation experiments, our objective was to assess the impact of integrating the latent alignment and attention modules on the prediction of drug response concentrations. Table 3 presents a comparison of the effects of integrating the individual modules on the prediction of drug response concentrations. The results clearly demonstrate that both the latent alignment and attention modules contribute to improved predictions. The latent alignment and attention modules significantly improve predictions, outperforming the baseline model, with MSE = 1.1333, F1-score = 0.5342, and AUROC = 0.5776. The latent alignment module only outperforms the baseline model in terms of the MSE, F1-score, and AUROC by 0.2375, 4.84%, and 6.1%, respectively. Similarly, adding the attention module yields improvements in these metrics, with differences of 0.1899, 2.88%, and 2.84%, respectively.

After integrating the latent alignment and attention modules into our model, we observed significant improvements in its ability to learn and integrate information from diverse omics data sources. The improved performance can be attributed to our model’s ability to discern the genes that truly influence drug response. This discernment is achieved through the utilization of optimizing integration strategies, enabling our model to learn the interrelationships among diverse omics datasets and the genetic information they contain. As a result, this acquired knowledge is seamlessly incorporated into the process of predicting drug responses.

### 3.4. Case Study on the Interpretability of the Model

After integrating latent alignment and attention modules into our model, we observed significant enhancements in its capacity to effectively gather and integrate information from various omics data sources. These advancements prompted us to explore the improved accuracy of our model in predicting drug response concentrations. The enhanced performance is primarily attributed to our model’s capability to identify the pivotal genes influencing drug responses. This ability stems from optimizing integration strategies, allowing our model to comprehend the complex relationships among diverse omics datasets and their genetic insights. Consequently, this acquired knowledge seamlessly contributes to the prediction of drug responses. We demonstrate our method in the following two experiments.

#### 3.4.1. Reactome Results Obtained by Integrating the Latent Alignment and Attention Modules

To determine whether the selected genes are truly associated with drug responses, first, we randomly selected a sample from the test dataset, which was a cell line sample of acute myeloid leukemia. Using our model, we predicted the response concentrations of 31 drugs for this sample and sorted the results. We found that the drug with the most effective predicted response was panobinostat, with a predicted value of −4.895, while the actual value was −5.600. The difference between these values was only 0.795. Finally, we identified the top 3% of genes in the model prediction of the patient’s response to panobinostat based on their contribution among all genes.

**Figure 3:**
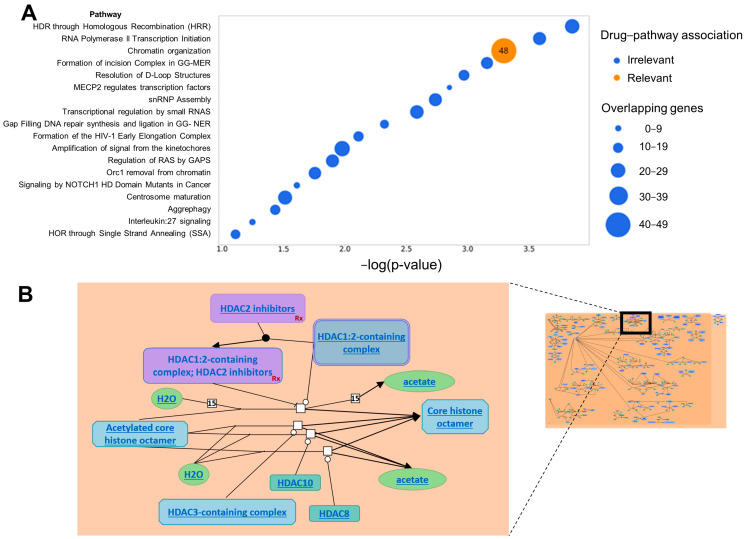
(**A**) Enriched signaling pathways and overlapping genes. The *x*-axis represents the significance of gene expression in the signaling pathways, where higher values indicate more significant expression of the important genes within the pathways. The *y*-axis represents the signaling pathways derived from the analysis of these important genes. The orange color represents the signaling pathways associated with panobinostat. The size of the circles corresponds to the overlap of important genes in the signaling pathways, with larger circles indicating a greater degree of overlap. (**B**) Chromatin organization signaling pathway and drug action site. By using Reactome, we found a pathway–gene network of chromatin organization signaling pathways and drug action sites. The upper right corner depicts the chromatin organization signaling pathway, while the left image illustrates the drug’s response location within this pathway.

The Reactome database is a peer-reviewed biological pathway database that provides comprehensive cellular processes [16]. Figure 3A illustrates the enriched signaling pathways obtained through pathway enrichment analysis using the identified top 3% important genes and the Reactome database for the specific pathway related to panobinostat, namely, the chromatin organization signaling pathway. The results of these 48 overlapping genes, demonstrating a significant −log(*p* value) of 3.29, can be observed in Supplementary Table S4. Additionally, to visualize the location and relationship of drug action within the signaling pathway associated with panobinostat, relevant information obtained from the Reactome dataset is displayed in Figure 3B. The drug enters through the HDAC2 inhibitor and affects the region indicated by the red line segment.

#### 3.4.2. Reactome Results by the Base Model

To validate the effectiveness of the latent alignment and attention modules incorporated into our model in improving the confidence of predictions, we conducted experiments using the base model on the same cell line sample afflicted with acute myeloid leukemia. Similarly, we predicted the drug concentrations of 31 drugs in response to this sample and ranked them accordingly. Panobinostat remained the most effective drug in terms of the predicted results, with a value of −4.110, deviating by 0.785 compared to the value of our model. In Supplementary Figure S3, it is evident that the ‘chromatin organization’ signaling pathway associated with panobinostat overlaps with 12 genes, as shown in Supplementary Table S5, and demonstrates a significantly low log(*p* value) of 1.19 × 10^−7^.

## 4. Discussion

In this study, we proposed a novel framework for accurately predicting drug response concentrations by effectively integrating multi-omics data. Our datasets comprise genomic-level information, including mutation and CNV data, as well as transcriptomic-level data encompassing methylation patterns and gene expression profiles. By integrating these diverse data types, our framework provides an interpretable prediction model with enhanced performance. Our results highlight the following important points. (i) Our approach outperforms two distinct multi-omics models. (ii) The latent alignment and attention modules significantly improve predictions, outperforming the baseline model, with MSE = 1.1333, F1-score = 0.5342, and AUROC = 0.5776. (iii) The case study demonstrates the most effective predicted response with clinical relevance.

Numerous machine learning models exist for the integration of multi-omics data [17,18]. Our approach specifically aligns omics features and captures correlation information among diverse omics datasets, thereby augmenting the overall performance of the model. To address feature misalignment, a latent alignment module was introduced to align different omics features in the same latent space after feature extraction, improving the integration results. An attention module was also incorporated to leverage information between omics data sources, capturing correlations and providing additional insights to enhance the model’s predictive capability. The performance of our model surpasses that of the reference study models [8,13], providing compelling evidence of its capability in drug response concentration prediction.

Moreover, ablation experiments confirm the significant impact of two key components: the latent alignment and attention modules. The latent alignment module facilitates the matching of multi-omics features during the training process, while the attention module captures the relationships among different omics datasets. Both modules significantly contribute to enhancing the performance of drug response prediction. By integrating both the attention and latent alignment modules, our final model achieves superior performance in predicting drug response concentrations, acquiring additional information, and improving the integration approach.

It is common to encounter missing values in omics data, originating from various factors [19]. To ensure robust predictions, we adopted a strategy that encompasses data preprocessing and leverages deep learning techniques for both feature extraction and prediction. By utilizing the learned target and considering the different omics datasets during feature extraction, we improved the similarity of samples across different data types, thereby enhancing the integration performance. The results clearly demonstrate that the improvement in the model performance is not solely attributed to including a larger amount of available information but also to the advantages of our integration approach.

To predict drug response concentrations, considering that the impact of various omics datasets on the prediction results may vary, we conducted an additional experiment. However, it is important to note that the negative impact of the mutation and CNV datasets does not necessarily imply that excluding these datasets would lead to better predictions of drug response. However, it does indicate that in our trained model, these two datasets contribute relatively less information and have a lesser impact on the final prediction results.

This study focused on predicting drug response concentrations [20,21], providing insights into both the effectiveness of patient drug responses and the necessary drug concentration for optimal inhibitory effects. In the case study, importantly, panobinostat is indeed the most effective drug in the actual response of the patient to the 31 drugs. Therefore, this prediction result is reasonable.

We observe consistent improvements in both the performance and interpretability of the model across various cancer types and their corresponding drug response predictions. The ability of our model to leverage genes directly associated with drug response further enhances the reliability of its predictions, even in the presence of diverse drug effects on patients. Overall, the experimental outcomes demonstrate the efficacy of our model architecture, particularly the attention and latent alignment modules, in predicting drug response concentrations. The successful integration of multi-omics data and improved interpretability highlight the potential of our model for enhancing drug response prediction in clinical settings.

## 5. Conclusions

In this study, we introduced the “Optimizing Integration Strategies” framework, designed to predict drug response concentrations accurately by integrating multi-omics data effectively. This framework incorporates genomic data such as mutations and CNV, alongside transcriptomic data including methylation patterns and gene expression levels. By combining these diverse data types, our framework offers an interpretable prediction model with enhanced performance.

Experimental results demonstrated the superiority of our “Optimizing Integration Strategies” framework compared to previous approaches for the same task. Further, ablation experiments confirmed the significant impact of two critical components: the latent alignment module, which aligns multi-omics features during training, and the attention module, which captures relationships among different omics data types. Both modules contributed to improved drug response prediction performance. These findings underscore the substantial advancements achieved by our proposed framework in predictive accuracy and its ability to leverage diverse omics datasets effectively.

Through comprehensive experimental analysis, we observed that methylation and gene expression datasets positively influenced drug response prediction, highlighting their significant contributions to our model. In contrast, mutation and CNV datasets had relatively smaller effects on the final prediction results, allowing for a more effective prioritization of relevant omics data for drug response prediction tasks.

Additionally, our investigation into the correlation between selected genes and actual drug response validated the reliability of our model’s predictions. Pathway enrichment analysis indicated that these identified important genes are directly involved in pathways relevant to the corresponding drugs.

By successfully integrating multiple omics data types, our model not only improves prediction accuracy but also enhances interpretability, facilitating the identification of key genetic factors influencing drug response. These results provide reliable insights for drug selection in medical decision-making and establish a robust framework for integrating diverse types of data.

## Figures and Tables

**Table 1:** Comparison of our model and reference methods for predicting drug response.

Method	MSE	F1-Score	AUROC
Sharifi-Noghabi H. et al. [8]	1.3556 ± 0.0062	0.5049 ± 0.0208	0.5151 ± 0.0037
Wang C et al. [10]	1.2455 ± 0.0101	0.5115 ± 0.0262	0.5277 ± 0.0078
Our model	1.1333 ± 0.0057	0.5342 ± 0.0162	0.5776 ± 0.0074

MSE: mean squared error; AUROC: area under the receiver operating characteristic curve.

**Table 2:** Performance differences among different combinations of omics datasets.

Combination	MSE	F1-Score	AUROC
Me–Ex–CNV–Mu	1.1333 ± 0.0057	0.5342 ± 0.0162	0.5776 ± 0.0074
Me–Ex–Mu	1.1592 ± 0.0041	0.5260 ± 0.0157	0.5632 ± 0.0077
Me–Ex–CNV	1.1960 ± 0.0203	0.4892 ± 0.0069	0.5539 ± 0.0108
Me–CNV–Mu	1.2170 ± 0.0135	0.4990 ± 0.0147	0.5535 ± 0.0056
Ex–CNV–Mu	1.2040 ± 0.0145	0.5162 ± 0.0280	0.5584 ± 0.0075
Me–Ex	1.2090 ± 0.0063	0.5104 ± 0.0183	0.5664 ± 0.0078
Me–CNV	1.3116 ± 0.0151	0.4733 ± 0.0159	0.5249 ± 0.0019
Me–Mu	1.2242 ± 0.0036	0.4935 ± 0.0153	0.5660 ± 0.0086
Ex–CNV	1.3006 ± 0.0047	0.4788 ± 0.0190	0.5103 ± 0.0107
Ex–Mu	1.2223 ± 0.0122	0.5123 ± 0.0276	0.5683 ± 0.0067
CNV–Mu	1.3209 ± 0.0059	0.5111 ± 0.0335	0.5221 ± 0.0074
Me	1.3660 ± 0.0080	0.4968 ± 0.0134	0.5177 ± 0.0098
EX	1.3397 ± 0.0016	0.4856 ± 0.0321	0.5120 ± 0.0056
CNV	1.3828 ± 0.0076	0.5012 ± 0.0118	0.5067 ± 0.0051
Mu	1.4201 ± 0.0257	0.5091 ± 0.0172	0.5040 ± 0.0031

**Table 3:** The overlapping genes in chromatin organization.

H2AX	KAT5	KAT14	ARID1A
ATF2	RBBP4	PRMT6	ELP6
H2AW	RUVBL2	SUZ12	SMARCA2
KMT2D	NSD1	NCOA2	GATAD2A
KDM5D	SAP130	SMARCC1	SMARCA4
KMT2A	EPC1	KDM2B	HCFC1
EHMT2	SAP18	PRMT1	TADA2A
EHMT1	SMYD3	EED	KANSL1
YEATS4	SUPT20H	MCRS1	KAT6B
ART3	JAK2	PRMT3	H2AC20
CHD4	MTA2	SETDB2	TADA3
SGF29	MTA3	ELP3	BRWD3

## Data Availability

The datasets used and analyzed during the current study are available from the corresponding author on reasonable request. The source code is available at the GitHub repository (https://github.com/Chei-YuanChi/Matster_Thesis). The raw data from the DepMap dataset (accessed on 10 August 2022) can be downloaded at https://drive.google.com/open?id=10O4lwyLxg5nLx7rB4i_--moxM6p2Gy1K&authuser=dpc0628%40gmail.com&usp=drive_fs (accessed on 10 August 2022).

## Supplementary Materials

The following supporting information can be downloaded at: https://www.mdpi.com/article/10.3390/jpm14070694/s1, Figure S1: The latent alignment module; Figure S2: Architecture of the attention module; Figure S3: Pathway analysis_based models; Table S1: The dataset sizes before and after preprocessing; Table S2: Individual drug response prediction results; Table S3: Drug-Index correspondence; Table S4: The overlapping genes in Chromatin Organization; Table S5: The overlapping genes in Chromatin Organization (Base model).
