# Supplementary material for: An Innovative Multi-Omics Model Integrating Latent Alignment and Attention Mechanism for Drug Response Prediction
Source: J Pers Med. 2024 Jun 27;14(7):694. doi: 10.3390/jpm14070694 (PMC11277895; doi:10.3390/jpm14070694)
Supplement: Supplementary file 1 [file jpm-14-00694-s001.zip › Supplementary Figure S1. The latent alignment module.pdf]

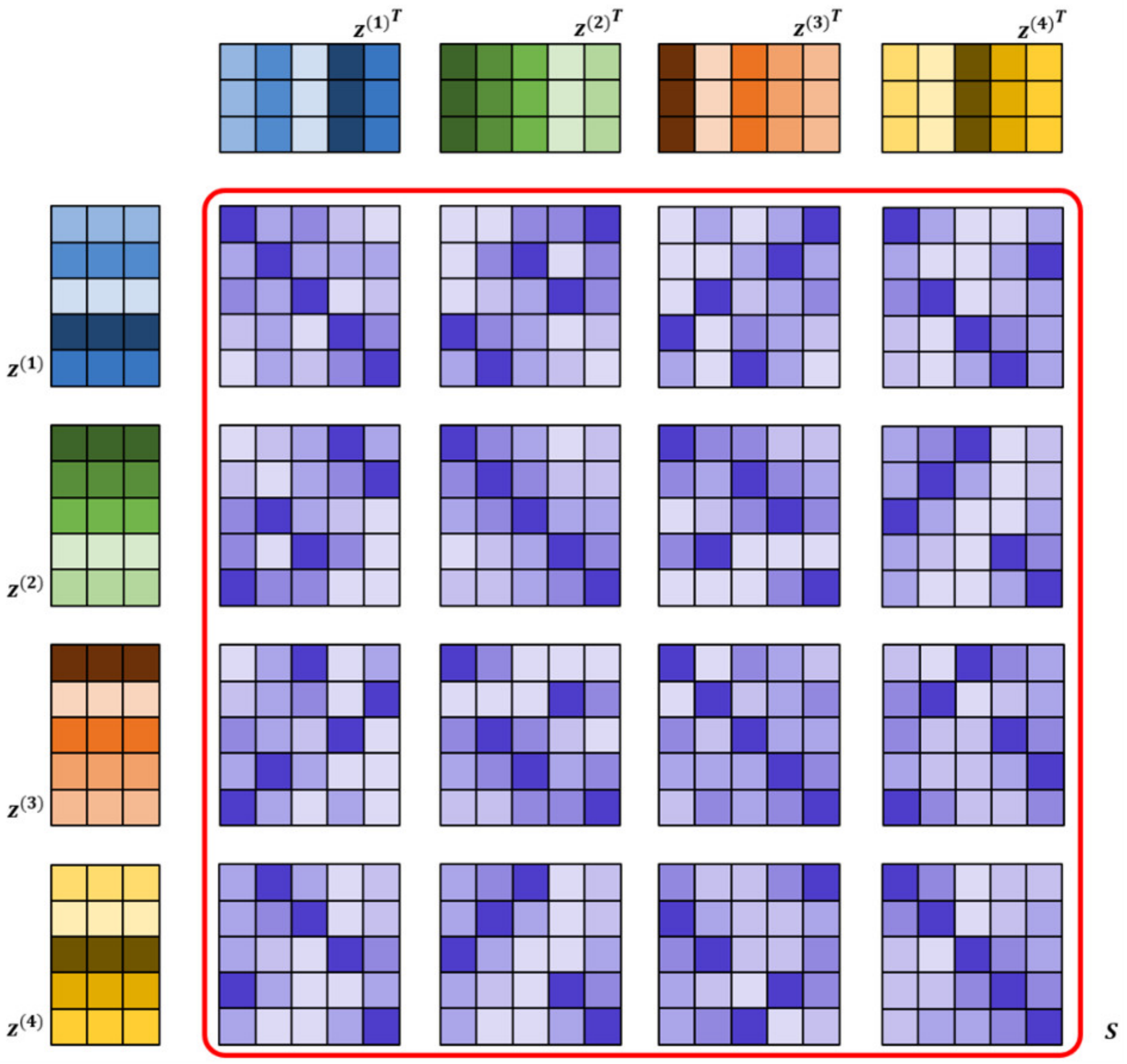

Supplementary Figure S1. Schematic representation for calculating sample similarity in the latent alignment module. This enables us to capture the differences between each omics data in the same sample in the latent space.
