# Supplementary material for: An Innovative Multi-Omics Model Integrating Latent Alignment and Attention Mechanism for Drug Response Prediction
Source: J Pers Med. 2024 Jun 27;14(7):694. doi: 10.3390/jpm14070694 (PMC11277895; doi:10.3390/jpm14070694)
Supplement: Supplementary file 1 [file jpm-14-00694-s001.zip › Supplementary Figure S2. Architecture of the attention module.pdf]

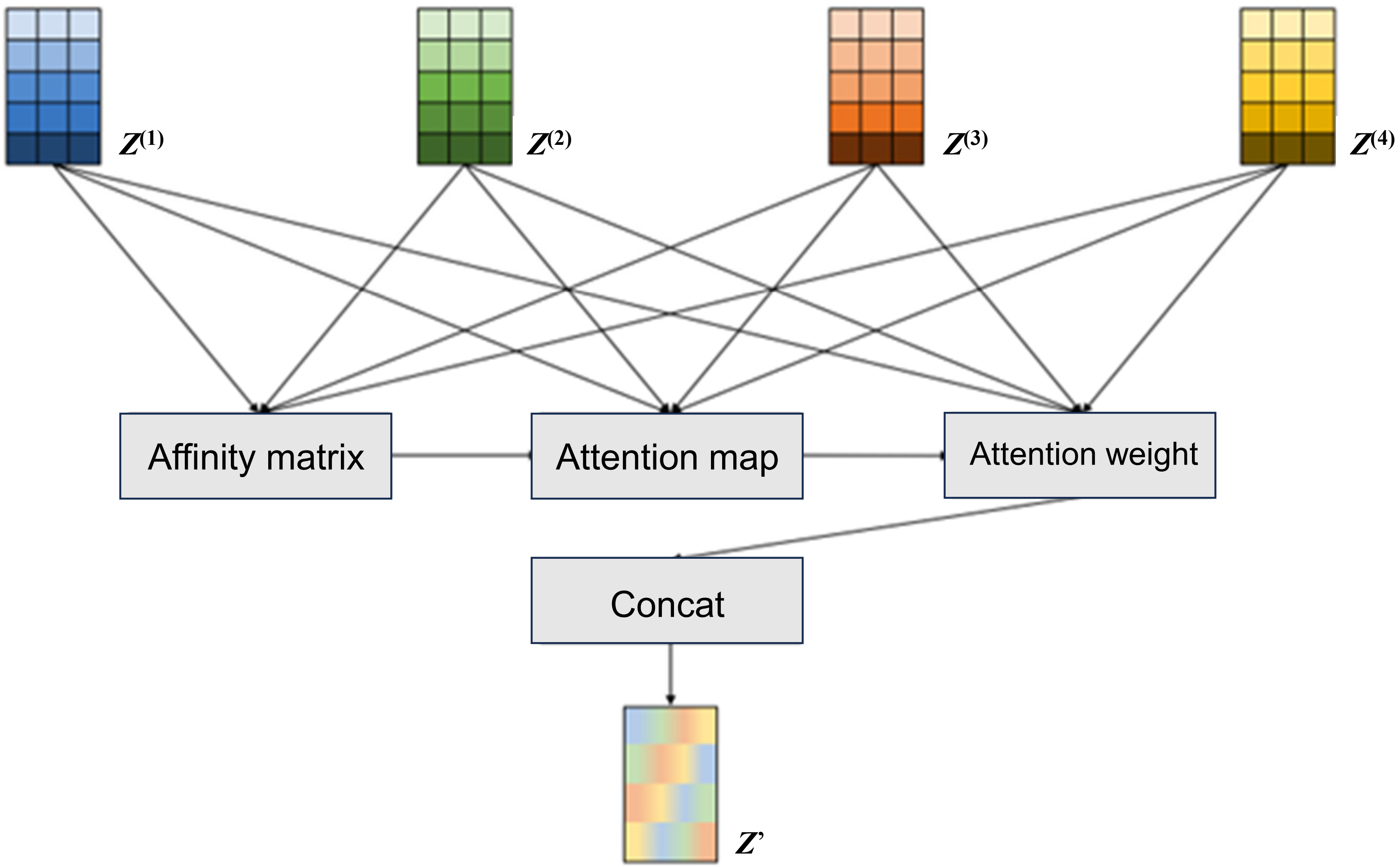


**Supplementary Figure S2.** The architecture of the attention module. The module incorporates information exchange between omics data through learnable matrices to learn the affinity matrices for each pair of omics data.
