# Supplementary material for: An Innovative Multi-Omics Model Integrating Latent Alignment and Attention Mechanism for Drug Response Prediction
Source: J Pers Med. 2024 Jun 27;14(7):694. doi: 10.3390/jpm14070694 (PMC11277895; doi:10.3390/jpm14070694)
Supplement: Supplementary file 1 [file jpm-14-00694-s001.zip › Supplementary Figure S3. Pathway abalysis_based models.pdf]

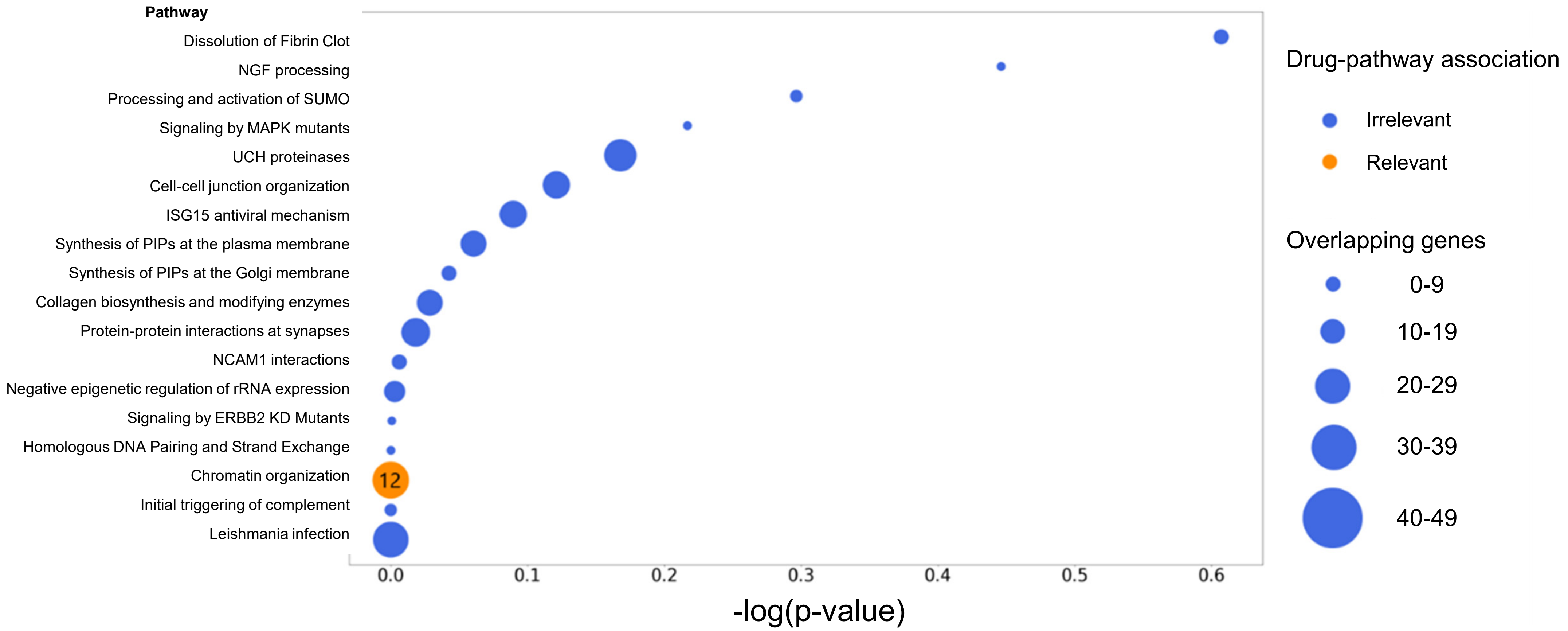

Supplementary Figure S3. Enriched signaling pathways and overlapping genes. The x-axis represents the significance of gene expression in the signaling pathways, where higher values indicate more significant expression of the important genes within the pathways. The y-axis represents the signaling pathways derived from the analysis of these important genes. The orange color represents the signaling pathways associated with Panobinostat. The size of the circles corresponds to the overlap of important genes in the signaling pathways, with larger circles indicating a greater degree of overlap.
