# Supplementary material for: An Innovative Multi-Omics Model Integrating Latent Alignment and Attention Mechanism for Drug Response Prediction
Source: J Pers Med. 2024 Jun 27;14(7):694. doi: 10.3390/jpm14070694 (PMC11277895; doi:10.3390/jpm14070694)
Supplement: Supplementary file 1 [file jpm-14-00694-s001.zip › Supplementary Table S1 The dataset sizes before and after preprocessing.pdf]

Supplementary Table S1. The dataset sizes before and after preprocessing

Datasets	Cell Line Samples		Number of Genes	
	Before	After	Before	After
Mutation	1,701	543	446	223
CNV	996	543	21,840	21,840
Methylation	833	543	20,219	19,412
Expression	1,406	543	19,160	19,144
IC50(log)	964	543	331	31

CNV: Copy number variation; IC50: Half maximal inhibitory concentration.
