# Supplementary material for: An Innovative Multi-Omics Model Integrating Latent Alignment and Attention Mechanism for Drug Response Prediction
Source: J Pers Med. 2024 Jun 27;14(7):694. doi: 10.3390/jpm14070694 (PMC11277895; doi:10.3390/jpm14070694)
Supplement: Supplementary file 1 [file jpm-14-00694-s001.zip › Supplementary Table S2. Individual drug response prediction results.pdf]

Supplementary Table S2. Individual drug response prediction results.

Drug	MSE	Drug	MSE
Piplartine	0.3735±0.0217	Foretinib	1.1665±0.0095
Tenovin-6	0.4187±0.0212	AR-42	0.5277±0.0078
CPI-613	0.4462±0.0281	LL-Z1640-2	1.2176±0.0410
Fingolimod	0.4656±0.0088	Dacinostat	1.3801±0.0819
LDN193189	0.4915±0.0095	IMD-0354	1.4158±0.0082
JW-7-24-1	0.5215±0.0209	AST-1306	1.4226±0.0366
ACY-1215	0.5501±0.0180	CAY10603	1.4456±0.0185
CI-1033	0.5548±0.0124	Anchusin	1.4635±0.0387
Vorinostat	0.5881±0.0372	CUDC-101	1.5072±0.0781
KIN001-204	0.6042±0.0006	Belinostat	1.5925±0.0254
CD532	0.7026±0.0286	GSK1059615	1.8074±0.0485
PF-00299804	0.7604±0.0017	Doxorubicin	1.9306±0.0529
AR-12	0.8135±0.0209	Dimethyloxalylglycine	2.0932±0.0913
TW-37	0.9769±0.0327	Obatoclax	2.2830±0.1293
Trichostatin A	1.0799±0.0240	Mitomycin-C	2.7332±0.0679
Panobinostat	1.1467±0.1032		

MSE: Mean squared error.
