# Supplementary material for: An Innovative Multi-Omics Model Integrating Latent Alignment and Attention Mechanism for Drug Response Prediction
Source: J Pers Med. 2024 Jun 27;14(7):694. doi: 10.3390/jpm14070694 (PMC11277895; doi:10.3390/jpm14070694)
Supplement: Supplementary file 1 [file jpm-14-00694-s001.zip › Supplementary Table S3. Drug-Index correspondence.pdf]

Supplementary Table S3. Drug-Index Correspondence.

Index	Drug	Index	Drug
1	Vorinostat	17	Belinostat
2	TW-37	18	CAY10603
3	LL-Z1640-2	19	Foretinib
4	Piplartine	20	Tenovin-6
5	Doxorubicin	21	CI-1033
6	Mitomycin-C	22	PF-00299804
7	KIN001-204	23	GSK1059615
8	Dimethyloxalylglycine	24	AST-1306
9	PDK1 inhibitor AR-12	25	CPI-613
10	Anchusin	26	Trichostatin A
11	Obatoclax	27	Panobinostat
12	Dacinostat	28	IMD-0354
13	JW-7-24-1	29	CD532
14	ACY-1215	30	LDN193189
15	AR-42	31	Fingolimod
16	CUDC-101		
