# Supplementary material for: An Innovative Multi-Omics Model Integrating Latent Alignment and Attention Mechanism for Drug Response Prediction
Source: J Pers Med. 2024 Jun 27;14(7):694. doi: 10.3390/jpm14070694 (PMC11277895; doi:10.3390/jpm14070694)
Supplement: Supplementary file 1 [file jpm-14-00694-s001.zip › Supplementary Table S4. The overlapping genes in Chromatin Organization.pdf]

Supplementary Table S4. The overlapping genes in Chromatin Organization

Gene list			
H2AX	KAT5	KAT14	ARID1A
ATF2	RBBP4	PRMT6	ELP6
H2AW	RUVBL2	SUZ12	SMARCA2
KMT2D	NSD1	NCOA2	GATAD2A
KDM5D	SAP130	SMARCC1	SMARCA4
KMT2A	EPC1	KDM2B	HCFC1
EHMT2	SAP18	PRMT1	TADA2A
EHMT1	SMYD3	EED	KANSL1
YEATS4	SUPT20H	MCRS1	KAT6B
ART3	JAK2	PRMT3	H2AC20
CHD4	MTA2	SETDB2	TADA3
SGF29	MTA3	ELP3	BRWD3
