# Supplementary material for: An Innovative Multi-Omics Model Integrating Latent Alignment and Attention Mechanism for Drug Response Prediction
Source: J Pers Med. 2024 Jun 27;14(7):694. doi: 10.3390/jpm14070694 (PMC11277895; doi:10.3390/jpm14070694)
Supplement: Supplementary file 1 [file jpm-14-00694-s001.zip › Supplementary Table S5. The overlapping genes in Chromatin Organization (Base model).pdf]

Supplementary Table S5. The overlapping genes in Chromatin Organization (Base model).

Gene list			
H2AX	MCRS1	SUPT20H	H2BC12
ARID5B	SMARCA2	MECOM	CHD4
ART3	SMARCE1	SETD6	JAK2
